# Viscoelastic Hemostatic Assays are Associated With Mortality and Blood Transfusion in a Multicenter Cohort

**DOI:** 10.1016/j.acepjo.2024.100042

**Published:** 2025-01-24

**Authors:** Shyam Murali, Eric Winter, Nicolas M. Chanes, Allyson M. Hynes, Madhu Subramanian, Alison A. Smith, Mark J. Seamon, Jeremy W. Cannon

**Affiliations:** 1 Division of Traumatology, Surgical Critical Care, and Emergency Surgery, University of Pennsylvania, Philadelphia, Pennsylvania, USA; 2 Division of Trauma and Surgical Critical Care, Grand View Health, Sellersville, Pennsylvania, USA; 3 Perelman School of Medicine, University of Pennsylvania, Philadelphia, Pennsylvania, USA; 4 Department of Surgery, University of Colorado School of Medicine, Aurora, Colorado, USA; 5 Department of Emergency Medicine, University of New Mexico, Albuquerque, New Mexico, USA; 6 Department of Surgery, University of New Mexico, Albuquerque, New Mexico, USA; 7 Division of Acute Care Surgery, Johns Hopkins School of Medicine, Baltimore, Maryland, USA; 8 Department of Surgery, Louisiana State University Health Sciences Center, University Medical Center New Orleans, New Orleans, Louisiana, USA

**Keywords:** AcoTS, acute coagulopathy of trauma shock, coagulopathy, ROTEM, TEG, thromboelastography, trauma-induced coagulopathy

## Abstract

**Objectives:**

Trauma-induced coagulopathy (TIC) carries significant risks, including increased mortality. Traditional TIC definitions rely on laboratories that result slowly and do not highlight therapeutic targets. We hypothesized that a TIC score, based on thromboelastography (TEG) and rotational thromboelastometry (ROTEM), collectively termed viscoelastic hemostatic assays, is associated with in-hospital mortality and packed red blood cell (pRBC) transfusion.

**Methods:**

This retrospective cohort study used a database of adult patients undergoing institutional massive transfusion at seven level 1 trauma centers (2013-2018). A “TIC score” was developed, with 1 point assigned for abnormal TEG R-time (≥ 9 min) or ROTEM clot time (≥ 80 sec), ɑ-angle (< 65^o^), or maximum amplitude (< 55 mm). TIC+ patients (TIC score 1-3) were compared with TIC− patients (TIC score 0). TIC Score composition and abnormal cutoff values were adjusted to investigate optimal weighting and thresholds. Multiple logistic and negative binomial regression was used to control confounders while evaluating the association between abnormal TIC values, in-hospital mortality, and 24-hour pRBC transfusion.

**Results:**

Of 1499 patients in the final analysis, 591 (39.4%) were TIC+. Each 1-point increase in TIC score was associated with a 53% increase in the odds of mortality (odds ratio [OR], 1.53, 95% CI, 1.33-1.76, *P* < .001) and a 25% increase in pRBC transfusion volumes (incidence rate ratio, 1.25, 95% CI, 1.16-1.34, *P* < .001). Abnormal maximum amplitude was associated with both mortality (OR 1.50, 95% CI, 1.03-2.19, *P* = .034) and pRBC transfusion volumes (*P* < .001), whereas abnormal ɑ-angle was associated with mortality (OR, 1.59, 95% CI, 1.09-2.32, *P* = .015). The unequal weighting of TIC score components and adjustments to normal/abnormal cutoff thresholds were maintained but did not improve the model’s predictive power.

**Conclusion:**

A viscoelastic hemostatic assay-based TIC score is independently associated with mortality and pRBC transfusion volumes. This association persists with unequal weighting and adjustment of normal/abnormal cutoff thresholds of TEG components.

**The Bottom Line**

In trauma patients, a simple scoring system based on abnormalities in thromboelastography and rotational thromboelastometry can predict the risk of death and the need for blood transfusions. Specifically, each 1-point increase in the trauma-induced coagulopathy score was associated with a 53% increase in the risk of death and a 25% increase in the volume of packed red blood cells needed. These findings suggest that the trauma-induced coagulopathy score may be valuable for identifying high-risk trauma patients in both a clinical setting during initial treatment and in future research endeavors seeking to improve outcomes in these patients.

## 1 Introduction

### 1.1 Background

Approximately 25% to 35% of all trauma patients develop acute coagulopathy that impacts their clinical course.^1^ These patients with coagulopathy patients are at a 3-fold higher risk of death compared with trauma patients without coagulopathy.^2^ Despite awareness of this increased mortality and improvements in damage control resuscitation, the etiology of coagulopathy in trauma has not been fully elucidated and is likely multifactorial.^3^ Currently, the management of trauma-induced coagulopathy (TIC) relies on standardized treatment protocols that do not address the nature of the hemostatic deficits but rather provide blood products in proportions that approach the composition of whole blood.^4^

### 1.2 Importance

Conventional coagulation assays (CCAs) such as partial thromboplastin time, prothrombin time, international normalized ratio, and fibrinogen analyze only specific factors involved in clotting, in isolation, and have long turnaround times.^5^ On the other hand, viscoelastic hemostatic assays (VHAs), such as thromboelastography (TEG) and rotational thromboelastometry (ROTEM), on whole blood samples spiked with various reagents provide a rapid assessment of global clot formation and dissolution, including contributions of both cellular (namely platelets and endothelial cells) and humoral components.^6^^,^^7^ In recent years, VHAs have become a more popular adjunct in the management of trauma patients as they can be used to guide the transfusion of blood products in real time.^7^^,^^8^ These tests may offer greater specificity for identifying patients likely to benefit from transfusion, and their use may also decrease unnecessary exposure to allogeneic blood products.^7^^,^^9^, ^10^, ^11^, ^12^, ^13^, ^14^ Based on the available evidence, the Eastern Association for the Surgery of Trauma conditionally recommends using TEG/ROTEM to guide blood transfusion for patients with ongoing hemorrhage and concern for coagulopathy.^15^

### 1.3 Goals of this Investigation

Among the roles of VHAs in the early management of trauma patients, their ability to predict mortality or transfusion quantities may be useful for logistic and operational reasons. However, systematic reviews have called for more research on the appropriate use of TEG/ROTEM in trauma, and optimal guidelines to incorporate the test into routine trauma protocols have not been determined.^16^, ^17^, ^18^ There is still a need to establish a robust correlation between TEG measurements and patient outcomes to help facilitate more informed clinical decision-making and enhance patient care. We hypothesized that a simple TEG-based score would correlate closely with packed red blood cell (pRBC) transfusion quantities and mortality in trauma patients receiving massive transfusion protocol.

## 2 Methods

### 2.1 Design, Setting, and Selection of Subjects

We performed a secondary analysis of a retrospective dataset collected by Smith et al^19^ from 2013 to 2018.^19^ This multicenter data set enrolled consecutive adult patients who received massive transfusion protocol (MTP) at 7 level 1 trauma centers in the United States. Each trauma center obtained Institutional Review Board approval. The inclusion criteria for the dataset were adult trauma patients receiving MTP during the study period who had TEG or ROTEM performed as part of their care. Patients with missing or incomplete data were excluded.^19^ Of the 7 centers, 5 utilized TEG, and 2 utilized ROTEM; patient demographic characteristics among the subset with TEG compared with those with ROTEM were evaluated for significant differences. MTP activation criteria were defined by institution-specific protocols. Demographic information was recorded along with arrival vital signs, obtained in the emergency department. Injury severity score (ISS) and quantity of blood product transfusion were also recorded. Patients with missing transfusion information for any blood product were assumed to have received 0 units of that product. The Strengthening the Reporting of Observational Studies in Epidemiology guideline was used to ensure proper reporting of methods, results, and discussion (Table S1).

### 2.2 Measures/Outcomes

For this analysis, we converted abnormal hypocoagulable values for both TEG and ROTEM to a TIC score based on normal vs abnormal values (Table 1), using reference normal ranges from the manufacturer, cross-referenced with established treatment protocols.^20^ TEG parameters considered were reaction time (R-time; denoted in this study as “clot time” to match ROTEM nomenclature), alpha angle at ten minutes (α-angle), and maximum amplitude (MA) (Table 1 highlights key VHA terminology). For ROTEM, extrinsically activated ROTEM values were used to obtain clot time, α-angle, and MA. While TEG and ROTEM are not fully equivalent tests with interchangeable results and interpretations, Venema et al^21^ demonstrated a close correlation between TEG and ROTEM testing for α-angle and MA; therefore, for this analysis, these results were aggregated between the tests.^22^ Clot lysis at 30 minutes (LY30) test score, which represents the proportion of clot breakdown, was not reported for the majority of patients in our study, and therefore, was not used as part of this TIC score. In the initial analysis, referred to as the “primary TIC score,” each abnormal parameter was given an equal weighting (1 point each) with a maximum score of 3. Patients with a TIC score of 1, 2, or 3 were considered TIC+. This scheme was selected for its simplicity, objectivity, and easy interpretability at the point-of-care.

**Table 1:** Viscoelastic hemostatic assay terminology with normal/abnormal cutoff thresholds, key definitions, and covariates. When calculating the trauma-induced coagulopathy score, each abnormal TEG component value was assigned 1 point in the primary trauma-induced coagulopathy score. Additional VHA parameter Lysis-30 (measure of clot lysis at 30 minutes) was not included in this analysis because it was not reported for the majority of patients.

		Normal (0 Points)	Abnormal (1 point)	Definition
VHA terminology	Reaction time (TEG)	<8.9 min	≥9 min	Time from activation of the coagulation cascade to the point when clot starts to form. This portion of the curve generally represents the activity and function of clotting factors and endothelium.
	Clot Time (ROTEM)	<79 sec	≥80 sec	
	Alpha angle (α-angle)	≥ 65^o^	< 65^o^	Measures the robustness of clot formation, representing the function of clotting factors, fibrinogen, and platelets.
	Maximum amplitude	≥55 mm	< 55 mm	Measures the strength of the maximally formed clot, assessing the dynamic properties of fibrin and platelet bonding.
	EXTEM	A ROTEM assay that utilizes tissue factor as an activator, evaluating the function of the EXtrinsic pathway in clotting.		
Covariates	Age (y)			
	Injury mechanism (penetrating vs blunt)			
	Heart rate			
	Systolic blood pressure			
	Glasgow coma scale			
	Hospital facility			
	Injury severity score - ∗Used as a covariate in a subgroup analysis∗			

EXTEM, extrinsic pathway thromboelastometry; ROTEM, rotational thromboelastometry; TEG, thromboelastography; VHA, viscoelastic assays.

### 2.3 Data Analysis

Multiple logistic regression and multiple negative binomial regression were subsequently used to evaluate the association between increasing TIC score and hospital mortality or pRBC transfusion volume for the hospitalization, respectively. Key covariates, including patient age, injury mechanism (penetrating vs blunt), presenting vital signs (heart rate and systolic blood pressure, and Glasgow coma scale [GCS] score), and hospital facility, were employed to mitigate the potential confounding influence of these variables on outcome analysis. Model covariates were selected for statistical (eg, age and presenting vitals) and clinical significance (eg, injury mechanism and hospital facility) and to account for their influence on patient management.

In sensitivity analyses, the fundamental parameters of the baseline TIC score were then manipulated to determine whether any variations in parameter weighting or positivity thresholds might yield significant increases in predictive power. First, the TIC components (clot time, α-angle, and MA) were assigned different weights (up to 3 points) in the overall TIC score calculation. Second, TIC score cut points were changed by increasing or decreasing the “normal vs abnormal” threshold by multiples of the overall population standard deviation. As before, multiple regression was used to evaluate the association between the modified TIC scores and key outcome variables. A final subgroup analysis was performed after excluding patients with missing ISS scores and utilizing ISS scores as a covariate. All statistical analysis was performed using STATA 17.0 (College Station, Texas).

## 3 Results

**Figure 1:**
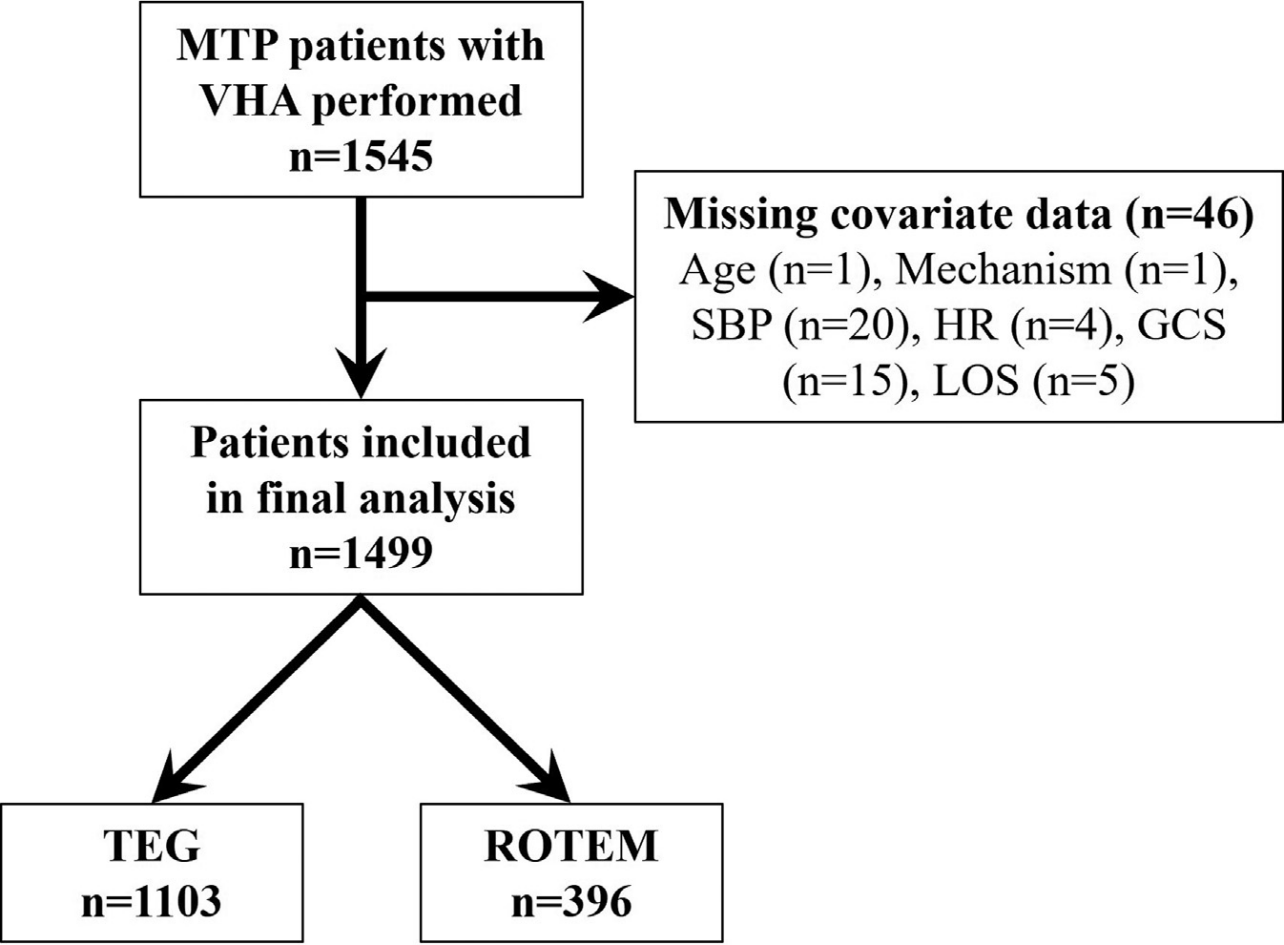
Flow diagram for the patient cohort. A total of 1499 patients were included in the final analysis. GCS, Glasgow coma scale; HR, heart rate; LOS, length of stay; MTP, massive transfusion protocol; ROTEM, rotational thromboelastometry; SBP, systolic blood pressure; TEG, thromboelastography; VHA, viscoelastic hemostatic assays.

From 2013 to 2018, a total of 1545 patients met the initial inclusion criteria. In the present work, 46 patients were subsequently excluded from the analysis due to missing covariate data (Fig 1). All remaining patients had complete demographic characteristics and outcome information.

In the remaining study cohort (Table 2), the mean age was 42.9 years, and the mean ISS was 29; 70.7% of patients were male. Age, systolic blood pressure, and GCS scores were significantly lower among TIC+ patients (all *P* < .001). The mean ISS was significantly higher among TIC+ patients (*P* < .001). TIC+ patients also received significantly higher transfusion volumes for all studied blood products (*P* < .001). One thousand one hundred three patients had TEG testing performed during their admission, whereas 396 patients had ROTEM performed. Using the previously described abnormal thresholds, 32 patients had abnormal R-times by TEG, and 102 patients had abnormal clot times by ROTEM. ɑ-Angle was abnormal in 422 patients, and MA was abnormal in 464 patients. Overall, 591 (39%) patients were found to be TIC+ by these baseline scoring criteria. Clot time was significantly longer, whereas α-angle and MA were significantly lower among TIC+ patients (all *P* < .001).

**Table 2:** Demographic characteristics, transfusion volumes, and TEG component values for the study population, comparing TIC− to TIC+.

		All patients			
		Overall n = 1499	TIC− n = 908	TIC+ n = 591	*P*-value
Patient characteristic	Age (y), mean (SD) or n (%)				<.001^a^
	Mean	42.9 (17.8)	43.2 (17.5)	40.1 (18.1)	
	18-45	907 (60.5)	524 (57.7)	383 (64.8)	
	46-65	389 (26.0)	251 (27.6)	138 (23.4)	
	>65	203 (13.5)	133 (14.7)	70 (11.8)	
	Sex, n (%)				.161
	Male	1060 (70.7)	630 (69.4)	430 (72.8)	
	Female	439 (29.3)	278 (30.6)	161 (27.2)	
	Race, n (%)				.458
	White	961 (64.1)	592 (65.2)	369 (62.4)	
	Black	423 (28.2)	252 (27.8)	171 (28.9)	
	Other/unknown	115 (7.7)	64 (7.1)	51 (8.6)	
	Injury type, n (%)				.016^a^
	Blunt	1021 (68.1)	640 (70.5)	381 (64.5)	
	Penetrating	478 (31.9)	268 (29.5)	210 (35.5)	
	Injury severity score, mean (SD) or n (%)				<.001^a^
	Mean	29.0 (15.4)	27.0 (14.3)	31.4 (16.3)	
	Unknown	428 (28.6)	333 (36.7)	95 (16.1)	
	1-8	48 (3.2)	28 (3.1)	20 (3.4)	
	9-25	437 (29.2)	255 (28.1)	182 (30.8)	
	26-75	586 (39.1)	292 (32.2)	294 (49.7)	
	Heart rate, mean (SD) or n (%)				.264
	Mean	101.9 (32.2)	101.1 (28.1)	103.0 (37.5)	
	0-50	59 (3.9)	22 (2.4)	37 (6.3)	
	51-100	662 (44.2)	438 (48.2)	244 (41.3)	
	101-150	707 (47.2)	421 (46.4)	286 (48.4)	
	>150	71 (4.7)	27 (3.0)	44 (7.4)	
	Systolic blood pressure, mean (SD) or n (%)				<.001^a^
	Mean	112.4 (39.6)	118.2 (36.0)	103.4 (43.0)	
	0-90	441 (29.4)	213 (23.5)	228 (38.6)	
	91-120	433 (28.9)	267 (29.4)	166 (28.1)	
	>120	625 (41.7)	428 (47.1)	197 (33.3)	
	Glasgow coma scale, mean (SD) or n (%)				<.001^a^
	Mean	10.1 (5.2)	11.2 (4.8)	8.5 (5.4)	
	3-8	586 (39.1)	272 (30.0)	314 (53.1)	
	9-12	118 (7.9)	74 (8.1)	44 (7.4)	
	13-15	795 (53.0)	562 (61.9)	233 (39.4)	
Transfusion volume	Packed red blood cells, mean (SD) or n (%)				<.001^a^
	Mean	9.2 (12.6)	6.7 (10.6)	12.9 (14.4)	
	0	350 (23.3)	250 (27.5)	100 (16.9)	
	1-5	427 (28.5)	318 (35.0)	109 (18.4)	
	6-10	238 (15.9)	138 (15.2)	100 (16.9)	
	10-20	292 (19.5)	136 (15.0)	156 (26.4)	
	>20	192 (12.8)	66 (7.3)	126 (21.3)	
Transfusion volume	Fresh frozen plasma, mean (SD) or n (%)				<.001^a^
	Mean	5.3 (8.9)	3.6 (7.5)	7.8 (10.3)	
	0	545 (36.4)	406 (44.7)	139 (23.5)	
	1-5	513 (34.2)	324 (35.7)	189 (32.0)	
	6-10	194 (12.9)	103 (11.3)	91 (15.4)	
	10-20	163 (10.9)	50 (5.5)	113 (19.1)	
	>20	84 (5.6)	25 (2.8)	59 (10.0)	
	Platelets, mean (SD) or n (%)				<.001^a^
	Mean	1.2 (2.8)	0.8 (1.9)	1.9 (3.7)	
	0	832 (55.5)	587 (64.6)	245 (41.5)	
	1-5	591 (39.4)	295 (32.5)	296 (50.1)	
	6-10	50 (3.3)	18 (2.0)	32 (5.4)	
	10-20	19 (1.3)	7 (0.8)	12 (2.0)	
	>20	7 (0.5)	1 (0.1)	6 (1.0)	
	Cryoprecipitate, mean (SD) or n (%)				<.001^a^
	Mean	0.6 (1.8)	0.3 (1.2)	1.0 (2.4)	
	0	1200 (80.1)	799 (88.0)	2.0 401	
	1-5	270 (18.0)	102 (11.2)	3.0 168	
	6-10	20 (1.3)	4 (0.4)	4.0 16	
	10-20	6 (0.4)	2 (0.2)	5.0 4	
	>20	3 (0.2)	1 (0.1)	6.0 2	
TIC score component	TEG clot Time				<.001^a^
	n (%)	1103 (73.6)	689 (75.8)	414 (70.0)	
	Normal Value	<8.9 min	<8.9 min	<8.9 min	
	Mean (SD)	4.3 (2.3)	3.5 (1.1)	5.4 (3.1)	
	0-8.9	1071 (97.1)	689 (100.0)	382 (92.3)	
	>8.9	32 (2.9)	0 (0.0)	32 (7.7)	
	ROTEM clot time				<.001^a^
	n	396 (26.4)	219 (24.2)	177 (30.0)	
	Normal Value	<79 s	<79 s	<79 s	
	Mean (SD)	77.7 (39.8)	64.4 (7.6)	94.2 (54.6)	
	0-79	294 (74.2)	219 (100.0)	75 (42.4)	
	>79	102 (25.8)	0 (0.0)	102 (57.6)	
	Alpha Angle				<.001^a^
	Normal Value	>65°	>65°	>65°	
	Mean (SD)	66.4 (11.9)	72.6 (3.5)	54.8 (13.8)	
	0-65	422 (28.2)	0 (0.0)	422 (71.4)	
	>65	1077 (71.8)	908 (100.0)	169 (28.6)	
	Maximum amplitude				<.001^a^
	Normal Value	>55 mm	>55 mm	>55 mm	
	Mean (SD)	57.3 (11.8)	63.9 (5.0)	47.1 (12.1)	
	0-55	464 (31.0)	0 (0.0)	464 (78.5)	
	>55	1035 (69.0)	908 (100.0)	127 (21.5)	

ROTEM, rotational thromboelastometry; SD, standard deviation; TEG, thromboelastography; TIC, trauma-induced coagulopathy.

a Indicates *P*-values <.05.

The ROTEM subgroup included slightly more female, white, and penetrating trauma victims (Table S2). Although these patients had similar ISS scores, the ROTEM group received more units of pRBC (8.1 vs 12.0), plasma (4.1 vs 8.6), and platelets (1.0 vs 1.7). However, there were no differences in TIC score elements between these subgroups.

### 3.1 Outcome Analysis Using Primary TIC Score

**Figure 2:**
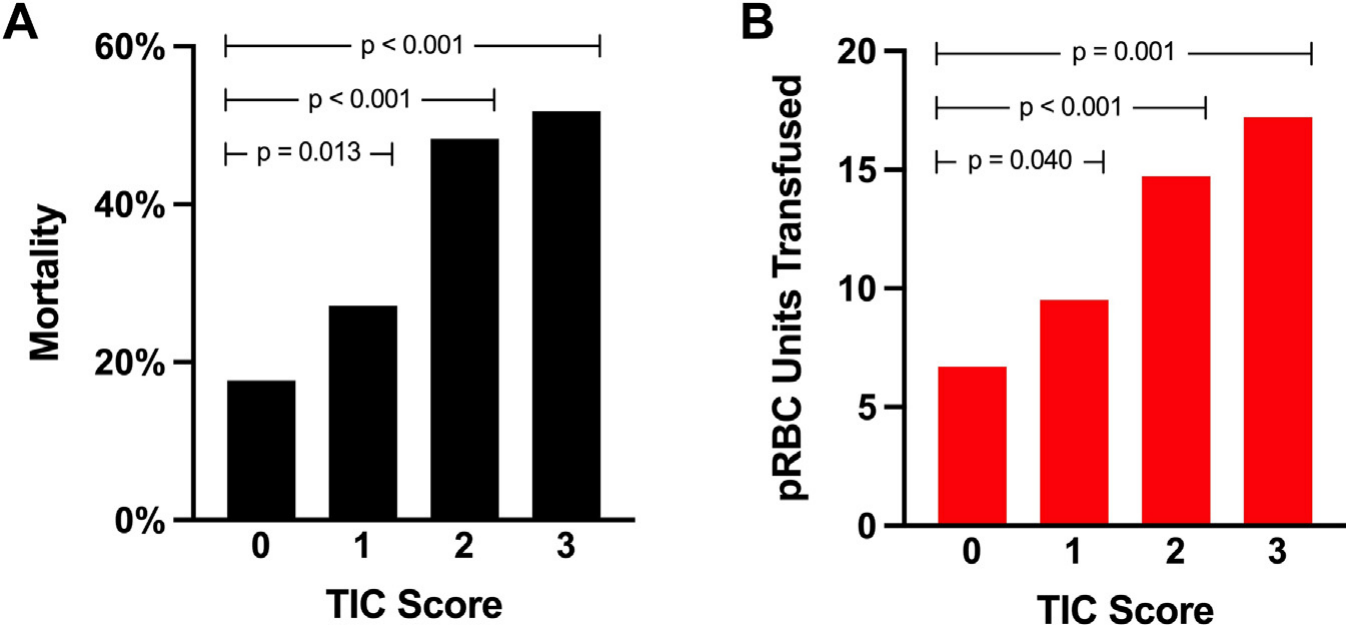
Mortality rate (A) and pRBC transfusion (B) using TIC score. pRBC packed red blood cell; TIC, trauma-induced coagulopathy.

**Figure 3:**
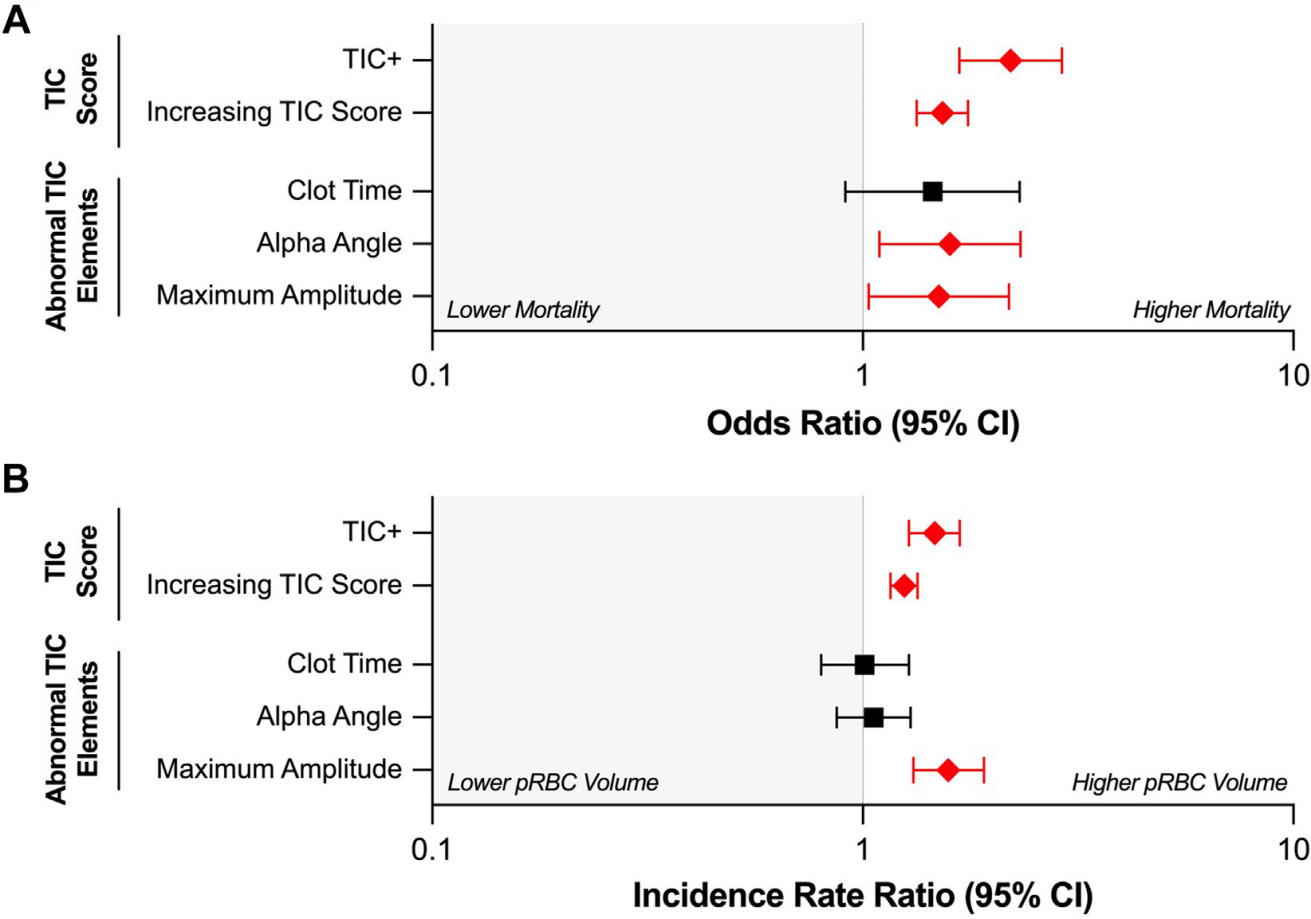
Forest plot for an odds ratio of mortality (A) and packed red blood cell (pRBC) transfusion (B) using TIC score and abnormal TIC elements. Red diamonds indicate statistically significant results. pRBC packed red blood cell; TIC, trauma-induced coagulopathy.

Increasing TIC score was significantly associated with both higher patient mortality and greater pRBC transfusion volume (Fig 2, Table S3). A 1-point increase in TIC score was associated with a 53% increase in mortality risk (OR, 1.53, 95% CI, 1.33-1.76, *P* < 0.001) and a 25% increase in average pRBC transfusion volume (incidence rate ratio [IRR], 1.25, 95% CI, 1.16-1.34, *P* < 0.001) (Fig 3, Table S4). When including covariates such as age, presenting vitals, and mechanism of injury, the TIC score has an area under the receiver operating characteristic (AUROC) value of 0.80 for predicting mortality. Abnormal clot time was not significantly associated with mortality or pRBC transfusion. However, abnormal α-angle was associated with a 59% increase in mortality risk (OR, 1.59, 95% CI, 1.09-2.32, *P* = .015). Similarly, the abnormal MA was correlated with a 50% increase in patient mortality (OR, 1.50, 95% CI, 1.03-2.19, *P* = .034) and a 58% increase in pRBC transfusion volume (IRR, 1.58, 95% CI, 1.31-1.91, *P* < .001).

### 3.2 Outcome Analysis Using Modified TIC Score Weights

TIC score components were subsequently assigned unequal weights, using a maximum score of 6 points. In these additional sensitivity analyses, increased TIC score was consistently found to be associated with increased risk of mortality and greater pRBC transfusion volume (all *P* < .001) (Table 3). No single model yielded a dramatically greater prediction of patient outcomes than the baseline model, as demonstrated using equivalent AUROC values of 0.80 for each mortality model permutation.

**Table 3:** Unequal weighting of TEG parameters in the TIC score. Model permutations 3 through 8 are compared against model permutation 2 for OR and incidence rate ratio to standardize the total possible TIC score.

		All patients								
		TIC component weight				TIC score prediction of outcome measure				
		Clot time	Alpha angle	Maximum amplitude	Total possible TIC score	Mortality			pRBC transfusion volume	
						OR (95% CI)	*P*-value	AUROC	IRR (95% CI)	*P*-value
Model permutation	1	1	1	1	3	1.53 (1.33-1.76)	<.001^a^	0.801	1.25 (1.16-1.34)	<.001^a^
	2	2	2	2	6	1.24 (1.16-1.33)	<.001^a^	0.801	1.12 (1.07-1.16)	<.001^a^
	3	1	2	3	6	1.20 (1.13-1.28)	<.001^a^	0.800	1.11 (1.07-1.14)	<.001^a^
	4	1	3	2	6	1.20 (1.13-1.28)	<.001^a^	0.801	1.10 (1.07-1.14)	<.001^a^
	5	2	1	3	6	1.23 (1.15-1.31)	<.001^a^	0.800	1.12 (1.08-1.16)	<.001^a^
	6	2	3	1	6	1.23 (1.15-1.31)	<.001^a^	0.801	1.11 (1.07-1.14)	<.001^a^
	7	3	1	2	6	1.26 (1.16-1.36)	<.001^a^	0.801	1.13 (1.08-1.17)	<.001^a^
	8	3	2	1	6	1.26 (1.16-1.36)	<.001^a^	0.801	1.11 (1.07-1.16)	<.001^a^
	9	TIC– vs TIC+			1	2.20 (1.67-2.90)	<.001^a^	0.798	1.47 (1.28-1.68)	<.001^a^

AUROC, area under the receiver operating characteristic; IRR, incidence rate ratio; OR, odds ratio; pRBC, packed red blood cell; TEG, thromboelastography; TIC, trauma-induced coagulopathy.

a Indicates *P*-values <.05.

### 3.3 Outcome Analysis Using Modified TIC Score Cut Points

The cutoff thresholds for normal and abnormal TIC score component values were then changed (Table S5). Again, across all model permutations, increased TIC score was consistently found to be associated with increased risk of mortality and greater pRBC transfusion volume (all *P* < .001). AUROC values were calculated for both the continuous baseline model (ie, TIC0 vs TIC1 vs TIC2 vs TIC3) and the dichotomous model (ie, TIC+ vs TIC–) (Fig S1). Similarly, no single model yielded a dramatically greater prediction of patient outcomes than the baseline model, as demonstrated by roughly equivalent AUROC values for each mortality model permutation.

### 3.4 Subgroup Analysis After Excluding Missing ISS Score

ISS score was missing in 428 (28.6%) of the study population; except for 3 of these cases, all the cases with missing ISS scores were from a single institution. Records with missing ISS scores were excluded and the analysis was performed, using ISS score as a covariate. A 1-point increase in TIC score was associated with a 46% increase in mortality risk (OR, 1.46, 95% CI, 1.26-1.69, *P* < .001) and a 21% increase in average pRBC transfusion volume (IRR, 1.21, 95% CI, 1.13-1.29, *P* < .001) (Fig S2, Table S6). None of the individual VHA components had correlations with mortality. Abnormal MA was correlated with a 49% increase in pRBC transfusion volume (IRR, 1.49, 95% CI, 1.24-1.79, *P* < .001).

## 4 Limitations

Although our study employed a large sample size of severely injured patients (ISS score 29 and GCS score 10) recruited from multiple hospitals, some noteworthy limitations of our study bear further discussion. As described in a previous analysis of these results by Smith et al^19^, the retrospective nature of our analysis could introduce bias into the results. Although 6-hour mortality may be the optimal outcome for hemorrhage control trials, we were limited by the existing dataset that only included hospital mortality.^23^ Additionally, we were unable to identify the exact cause of death from this dataset and therefore could not exclude patients with nonhemorrhage-related deaths. Anticoagulation and antiplatelet status were also not recorded in the original dataset and, therefore, could not be used in this analysis. Although some anticoagulants can be monitored with TEG, others such as warfarin have been shown to have a poor correlation with TEG parameters.^24^^,^^25^

Injury severity was not used as a covariate in the initial analysis given that many patients (n = 428) were missing this data. However, in a subgroup analysis that excluded patients with missing ISS and included ISS as a covariate, the findings were similar to the primary investigation.

LY30 test score was not reported for the majority of patients in our study, and although it is a crucial piece of information in TEG and ROTEM, we were unable to include it in our TIC score. Future research should include all pieces of information provided by VHAs. LY30 test score, however, would have a bidirectional impact on coagulation, indicating hypercoagulability when the LY30 test score is too low and hyperfibrinolysis when the LY30 score is high.

## 5 Discussion

Viscoelastic tests, including both TEG and ROTEM, have become a staple of modern trauma care, especially for patients with hemorrhagic shock, traumatic brain injury, or antiplatelet/anticoagulant use.^26^ We performed a secondary analysis of a multicenter database with nearly 1500 patients. The most important finding of this study was a strong association between increasing TEG abnormalities and hospital mortality (53% increase per point increase in TIC score) and pRBC transfusion volume (25% increase per point increase). Abnormal MA was associated with both mortality and pRBC transfusion volumes, whereas abnormal α-angle was associated with mortality. Altering the weighting of each component maintained the association with the primary TIC score but did not meaningfully change the predictive ability of the model. Previously published literature to identify an alternative weighting scheme was unavailable to identify an optimal assignment of point values a priori. Therefore, we assessed all the combinations of point values possible for potential differences in outcomes. Similarly, increasing or decreasing the cutoff thresholds for abnormality did not change the predictive ability of the model. Our analysis of TEG vs ROTEM also revealed center-level variations in demographic characteristic, vital signs, and initial empiric resuscitation with blood products. Despite similar injury severity scores, the latter may have been influenced by the higher proportion of penetrating injury patients who are known to have a higher burden of injury at similar ISS score levels compared with blunt injury counterparts.^27^ Furthermore, these differences may have been driven by institution-specific MTP activation criteria. However, there were no significant differences identified in α-angle and MA between TEG and ROTEM.

Anticipating the need for a massive transfusion is clinically valuable as it can facilitate early and effective communication with the blood bank, allowing for ample time to prepare blood products.^28^ Our study is consistent with previous literature showing the predictive ability of TEG and ROTEM parameters for mortality and pRBC transfusion. In a large retrospective cohort study, rapid-thromboelastography-activated clotting time and α-angle emerged as superior predictors of massive RBC transfusion, surpassing CCAs.^29^ The diagnosis and treatment of TIC study investigators showed that the fibrin-based extrinsically activated test with tissue factor and the platelet inhibitor cytochalasin D maximum clot firmness and the extrinsically activated test with tissue factor maximum clot firmness were independently associated with RBC transfusion and early mortality, respectively.^30^ Other smaller studies have shown similar associations between clot strength (which, in 1 study, had a very high predictive power for MT-related death, AUROC value of 0.93) and pRBC transfusion and mortality.^31^, ^32^, ^33^

Elevated R-time (clot time) and LY30 test score have been associated with an increased risk of hospital mortality; in 1 retrospective review, clot time > 6 minutes (indicative of a hypocoagulable state) was an independent risk factor for death (odds ratio of 16) in patients with pelvic trauma.^34^^,^^35^ Savage and colleagues proposed a single TEG value at admission, the ratio between the maximum amplitude and R-time, which would reflect both the hypercoagulable (higher MA-R ratios) and hypocoagulable (lower MA-R ratios) states of TIC.^36^ They showed that the MA-R ratios were inversely proportional to mortality when their dataset was grouped using quartiles (MA-R ratio of < 14.2, 14.2-19.5, 19.5-23.8, > 23.8). A subsequent study using the pragmatic randomized optimal platelet and plasma ratios dataset identified a “CRITICAL: MA-R” ratio cut point of ≤ 11 by Youden’s index; this cut point had significant associations with mortality in both penetrating and blunt subgroups, as well as significant elevations in multiple proinflammatory cytokines.^37^^,^^38^

These associations have been put into practice and evaluated through small randomized controlled trials (RCTs). In 2016, Gonzalez et al^39^ published the results of a single-center RCT, comparing patients who were managed using an MTP goal, directed either by TEG or by CCAs. They found that mortality was significantly lower in the TEG group compared with CCA (19.6% vs 36.4%; overall mortality of 27.9%). In contrast, the multicenter implementing treatment algorithms for the correction of TIC RCT compared outcomes for patients who received an empiric massive hemorrhage protocol supplemented by therapy guided by either CCAs or VHAs.^4^ The investigators found no difference between groups in the proportion of patients who were alive and free of massive transfusion (10 or more units of pRBCs in the first 24 hours) and mortality at 28 and 90 days from randomization. However, the study enrolled a group of patients with a lower-than-expected prevalence of coagulopathy, and the authors found it unsurprising that coagulation monitoring did not alter the clinical outcome. Of note, the subgroup of patients with traumatic brain injury had lower 28-day mortality when their resuscitation was guided by VHAs.

The findings of our study support and strengthen the theory that TEG abnormalities can predict transfusion requirements and mortality. Furthermore, we propose that TEG/ROTEM abnormalities should be studied prospectively, either in isolation or in combination with other parameters, as part of a coagulopathy score. Similar to the age, blood tests, and comorbidities score, based upon our findings, a TIC score of ≥ 2 clearly identifies a patient at high risk of death from hemorrhage-associated coagulopathy.^40^ Future studies should seek to determine whether these patients would benefit from intensified efforts to identify and stop sources of ongoing hemorrhage and more aggressive reversal of coagulopathy.

The TIC score, assigning one point for each abnormal value, is associated with mortality and pRBC transfusion volumes. Individual VHA parameters, namely the α-angle and MA, are independently associated with hospital mortality. Future prospective studies should be performed to assess the impact of a VHA-based score on patient outcomes.

## Author Contributions

Literature search: SM, EW, NC, AH, MSu, AS

Study design: SM, EW, NC, AH, MSu, AS, MSe, JC

Data collection: SM, NC, AS, JC

Data analysis: SM, EW, AS, JC

Data interpretation: SM, EW, JC

Manuscript drafting: SM, NC, EW, JC

Critical Review: SM, EW, NC, AH, MSu, AS, MSe, JC

## Funding and Support

This work did not receive any funding from any sources.

## Conflict of Interest

Conflicts of Interest are documented in an attached supplement.
