# Supplementary material for: Viscoelastic Hemostatic Assays are Associated With Mortality and Blood Transfusion in a Multicenter Cohort
Source: J Am Coll Emerg Physicians Open. 2025 Jan 24;6(2):100042. doi: 10.1016/j.acepjo.2024.100042 (PMC11997675; doi:10.1016/j.acepjo.2024.100042)
Supplement: Supplementary Figure 1 and 2 and Supplementary Tables 1-6 [file mmc1.docx]

**Viscoelastic Hemostatic Assays are Associated**

**with Mortality and Blood Transfusion in a Multicenter Cohort**

*Supplemental Digital Content*

Shyam Murali, MD^1,2^, Eric Winter, MD, MBA^3^, Nicolas M. Chanes, MD^4^, Allyson M. Hynes, MD^5,6^, Madhu Subramanian, MD^7^, Alison A. Smith, MD, PhD^8^, Mark J. Seamon, MD^1^, *Jeremy W. Cannon, MD, SM^1^

^1^Division of Traumatology, Surgical Critical Care, and Emergency Surgery, Department of Surgery, Perelman School of Medicine at the University of Pennsylvania, Philadelphia, PA

^2^Division of Trauma and Surgical Critical Care, Grand View Health, Sellersville, PA

^3^University of Pennsylvania Health System, Philadelphia, PA

^4^Department of Surgery, University of Colorado School of Medicine, Aurora, CO

^5^Department of Emergency Medicine, University of New Mexico, Albuquerque, NM

^6^Department of Surgery, University of New Mexico, Albuquerque, NM

^7^Division of Acute Care Surgery, Johns Hopkins School of Medicine, Baltimore, MD

^8^Louisiana State University Health Sciences Center, University Medical Center, New Orleans, LA

*Corresponding Author

jeremy.cannon@pennmedicine.upenn.edu

(215) 662-7320

**
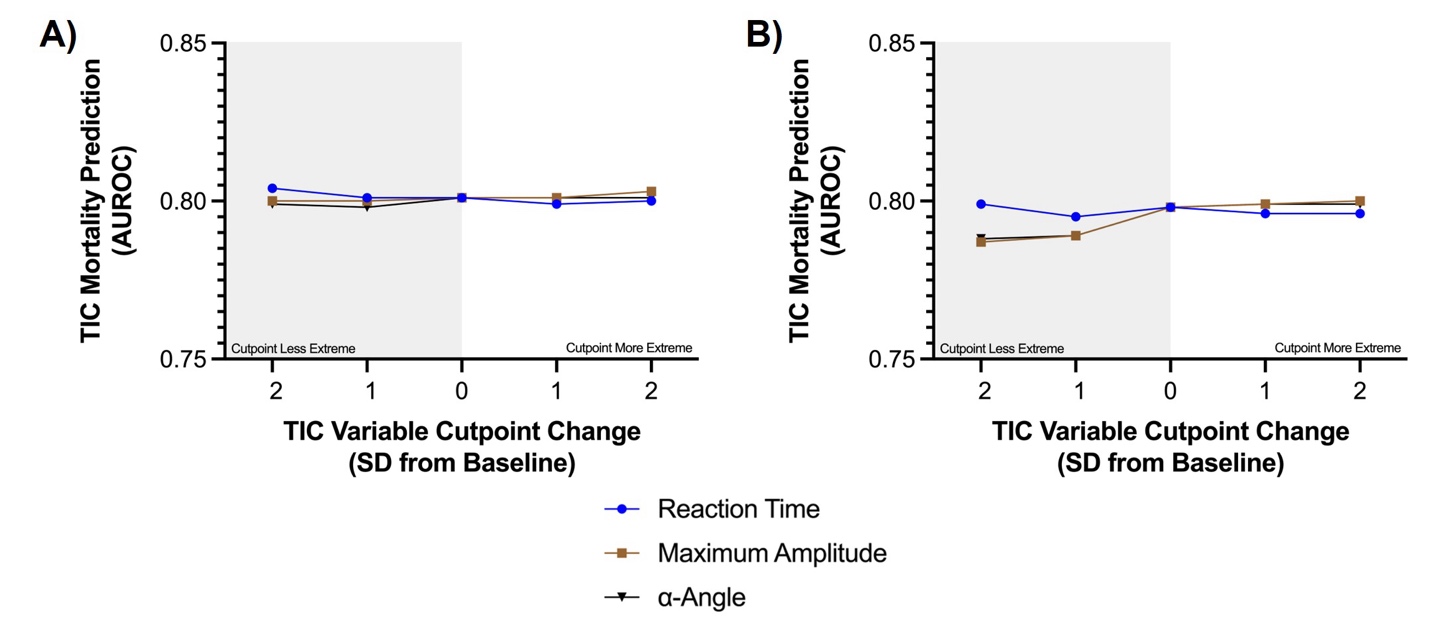
**

**Supplemental Digital Content Figure 1:** AUROC for TIC score based on alternative cut points for each component of the TEG score. There is minimal change in AUROC when using modified cut points both below and above the baseline for both the continuous model (i.e., TIC0 vs TIC1 vs TIC2 vs TIC3; panel A) and the dichotomous model (i.e., TIC+ vs TIC–; panel B).


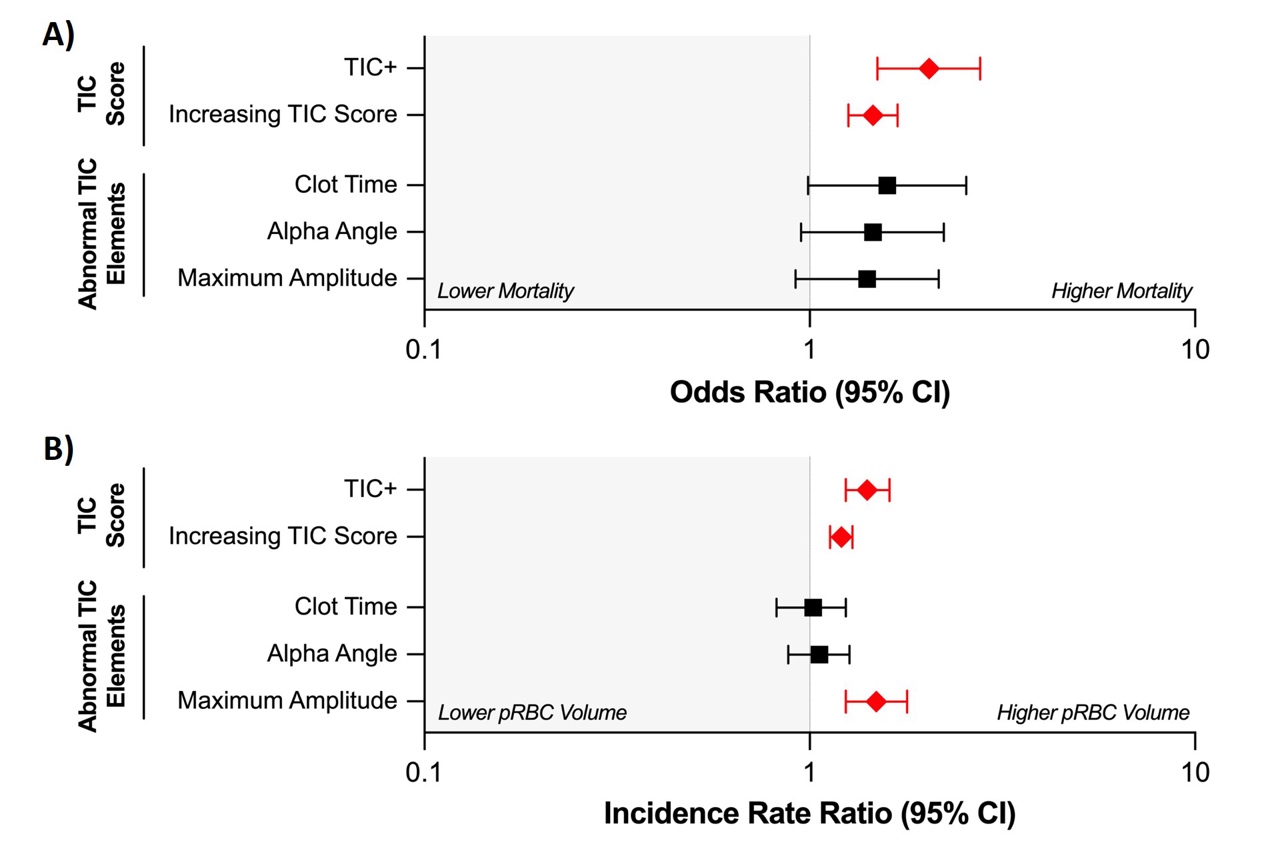


**Supplemental Digital Content Figure 2:** Forest plot for odds ratio of mortality (panel A) and pRBC transfusion (panel B) by TIC score and abnormal TIC elements, after excluding patients with missing ISS and utilizing ISS as a covariate. Red diamonds indicate statistically significant results.

**Supplemental Digital Content Table 1:** STROBE Statement—checklist of items that should be included in reports of observational studies

|  | Item No | Recommendation | Page  No |
| --- | --- | --- | --- |
| **Title and abstract** | 1 | (*a*) Indicate the study’s design with a commonly used term in the title or the abstract | 1 |
|  |  | (*b*) Provide in the abstract an informative and balanced summary of what was done and what was found | 4 |
| Introduction | | | |
| Background/rationale | 2 | Explain the scientific background and rationale for the investigation being reported | 6 |
| Objectives | 3 | State specific objectives, including any prespecified hypotheses | 7 |
| Methods | | | |
| Study design | 4 | Present key elements of study design early in the paper | 7 |
| Setting | 5 | Describe the setting, locations, and relevant dates, including periods of recruitment, exposure, follow-up, and data collection | 7 |
| Participants | 6 | (*a*) *Cohort study*—Give the eligibility criteria, and the sources and methods of selection of participants. Describe methods of follow-up  *Case-control study*—Give the eligibility criteria, and the sources and methods of case ascertainment and control selection. Give the rationale for the choice of cases and controls  *Cross-sectional study*—Give the eligibility criteria, and the sources and methods of selection of participants | 7 |
|  |  | (*b*) *Cohort study*—For matched studies, give matching criteria and number of exposed and unexposed  *Case-control study*—For matched studies, give matching criteria and the number of controls per case |  |
| Variables | 7 | Clearly define all outcomes, exposures, predictors, potential confounders, and effect modifiers. Give diagnostic criteria, if applicable | 8 |
| Data sources/ measurement | 8* | For each variable of interest, give sources of data and details of methods of assessment (measurement). Describe comparability of assessment methods if there is more than one group | 8 |
| Bias | 9 | Describe any efforts to address potential sources of bias | 8 |
| Study size | 10 | Explain how the study size was arrived at | 8 |
| Quantitative variables | 11 | Explain how quantitative variables were handled in the analyses. If applicable, describe which groupings were chosen and why | 7-8 |
| Statistical methods | 12 | (*a*) Describe all statistical methods, including those used to control for confounding | 8 |
|  |  | (*b*) Describe any methods used to examine subgroups and interactions | 8 |
|  |  | (*c*) Explain how missing data were addressed | 8 |
|  |  | (*d*) *Cohort study*—If applicable, explain how loss to follow-up was addressed  *Case-control study*—If applicable, explain how matching of cases and controls was addressed  *Cross-sectional study*—If applicable, describe analytical methods taking account of sampling strategy | 8 |
|  |  | (*e*) Describe any sensitivity analyses | 8 |

| Results | | | |
| --- | --- | --- | --- |
| Participants | 13* | (a) Report numbers of individuals at each stage of study—eg numbers potentially eligible, examined for eligibility, confirmed eligible, included in the study, completing follow-up, and analysed | 9 |
|  |  | (b) Give reasons for non-participation at each stage | 9 |
|  |  | (c) Consider use of a flow diagram | Fig1 |
| Descriptive data | 14* | (a) Give characteristics of study participants (eg demographic, clinical, social) and information on exposures and potential confounders | Table 2 |
|  |  | (b) Indicate number of participants with missing data for each variable of interest | Fig1 |
|  |  | (c) *Cohort study*—Summarise follow-up time (eg, average and total amount) |  |
| Outcome data | 15* | *Cohort study*—Report numbers of outcome events or summary measures over time |  |
|  |  | *Case-control study—*Report numbers in each exposure category, or summary measures of exposure |  |
|  |  | *Cross-sectional study—*Report numbers of outcome events or summary measures | 9 |
| Main results | 16 | (*a*) Give unadjusted estimates and, if applicable, confounder-adjusted estimates and their precision (eg, 95% confidence interval). Make clear which confounders were adjusted for and why they were included | 9 |
|  |  | (*b*) Report category boundaries when continuous variables were categorized |  |
|  |  | (*c*) If relevant, consider translating estimates of relative risk into absolute risk for a meaningful time period |  |
| Other analyses | 17 | Report other analyses done—eg analyses of subgroups and interactions, and sensitivity analyses | 10 |
| Discussion | | | |
| Key results | 18 | Summarise key results with reference to study objectives | 11 |
| Limitations | 19 | Discuss limitations of the study, taking into account sources of potential bias or imprecision. Discuss both direction and magnitude of any potential bias | 14 |
| Interpretation | 20 | Give a cautious overall interpretation of results considering objectives, limitations, multiplicity of analyses, results from similar studies, and other relevant evidence | 15 |
| Generalisability | 21 | Discuss the generalisability (external validity) of the study results | 14 |
| Other information | | | |
| Funding | 22 | Give the source of funding and the role of the funders for the present study and, if applicable, for the original study on which the present article is based | 7 |

*Give information separately for cases and controls in case-control studies and, if applicable, for exposed and unexposed groups in cohort and cross-sectional studies.

**Note:** An Explanation and Elaboration article discusses each checklist item and gives methodological background and published examples of transparent reporting. The STROBE checklist is best used in conjunction with this article (freely available on the Web sites of PLoS Medicine at http://www.plosmedicine.org/, Annals of Internal Medicine at http://www.annals.org/, and Epidemiology at http://www.epidem.com/). Information on the STROBE Initiative is available at www.strobe-statement.org.

**Supplemental Digital Content Table 2:** Analysis of TEG vs ROTEM patients, comparing patient characteristics, transfusion volumes, and TIC score components.

|  |  | **All Patients** | | | |
| --- | --- | --- | --- | --- | --- |
|  |  | Overall  n = 1499 | TEG  n = 1103 | ROTEM  n = 396 | p-value |
| **Patient Characteristic** | Age  Mean (SD) | 42.9 (17.8) | 42.3 (18.0) | 40.9 (17.2) | 0.183 |
|  | Sex, n (%)  Male  Female | 1060 (70.7)  439 (29.3) | 886 (78.5)  237 (21.5) | 194 (49.0)  202 (51.0) | **< 0.001** |
|  | Race, n (%)  White  Black  Other/Unknown | 961 (64.1)  423 (28.2)  115 (7.7) | 692 (62.7)  314 (28.5)  97 (8.8) | 269 (67.9)  109 (27.5)  18 (4.3) | **0.008** |
|  | Injury Type, n (%)  Blunt  Penetrating | 1021 (68.1)  478 (31.9) | 772 (70.0)  331 (30.0) | 249 (62.9)  147 (37.1) | **0.009** |
|  | ISS  Mean (SD) | 29 (15) | 29 (15) | 29 (16) | 0.420 |
|  | HR  Mean (SD) | 102 (32) | 100 (32) | 108 (33) | **< 0.001** |
|  | SBP  Mean (SD) | 112 (40) | 115 (40) | 106 (39) | **< 0.001** |
|  | GCS  Mean (SD) | 10 (5) | 10 (5) | 10 (5) | **0.009** |
| **Transfusion Volume** | Packed Red Blood Cells  Mean (SD) | 9.2 (12.6) | 8.1 (12.0) | 12.0 (13.7) | **< 0.001** |
|  | Fresh Frozen Plasma  Mean (SD) | 5.3 (8.9) | 4.1 (8.1) | 8.6 (10.2) | **< 0.001** |
|  | Platelets  Mean (SD) | 1.2 (2.8) | 1.1 (2.9) | 1.7 (2.4) | **< 0.001** |
|  | Cryoprecipitate  Mean (SD) | 0.6 (1.8) | 0.5 (1.7) | 0.7 (1.9) | 0.086 |
| **TIC Score Component** | Alpha Angle  Normal Value  Mean (SD)  0-65  > 65 | > 65°  66.4 (11.9)  422 (28.2)  1077 (71.8) | > 65°  66.6 (12.2)  295 (26.8)  808 (73.3) | > 65°  65.8 (10.9)  127 (32.1)  269 (67.9) | 0.247 |
|  | Maximum Amplitude  Normal Value  Mean (SD)  0-55  > 55 | > 55 mm  57.3 (11.8)  464 (31.0)  1035 (69.0) | > 55 mm  57.3 (12.5)  347 (31.5)  756 (68.5) | > 55 mm  57.3 (9.7)  117 (29.6)  279 (70.5) | 0.979 |

**Supplemental Digital Content Table 3:** Mortality rate and pRBCs transfused by TIC score.

|  |  | **TIC Score** | | | | | |
| --- | --- | --- | --- | --- | --- | --- | --- |
|  |  | Overall | 0 | 1 | 2 | 3 | p-value |
| **Metric** | Patient Count  n (%) | 1499 | 908  (60.7) | 247  (16.5) | 259  (17.3) | 85  (5.7) | NA |
|  | Mortality  n (%) | 397  (26.5) | 161  (17.7) | 67  (27.1) | 125  (48.3) | 44  (51.8) | **< 0.001** |
|  | pRBCs Transfused  mean units (SD) | 9.2  (12.6) | 6.7  (10.6) | 9.5  (11.9) | 14.7  (15.5) | 17.2  (15.4) | **< 0.001** |

**Supplemental Digital Content Table 4:** Predictive abilities of TIC score and abnormal TEG components on mortality and pRBC transfusion volumes. Effect on mortality is presented as odds ratio, while pRBC transfusion is presented as an incidence rate ratio.

|  |  | **Outcome Measure** | | | |  |
| --- | --- | --- | --- | --- | --- | --- |
|  |  | Mortality | | pRBC  Transfusion Volume | |  |
|  |  | OR  (95% CI) | p-value | IRR  (95% CI) | p-value | |
| **Predictive Factor** | TIC Score | 1.53  (1.33-1.76) | **< 0.001** | 1.25  (1.16-1.34) | **< 0.001** | |
|  | Clot Time Abnormal | 1.45  (0.91-2.31) | 0.116 | 1.01  (0.80-1.28) | 0.939 | |
|  | Alpha Angle Abnormal | 1.59  (1.09-2.32) | **0.015** | 1.06  (0.87-1.29) | 0.585 | |
|  | Maximum Amplitude Abnormal | 1.50  (1.03-2.19) | **0.034** | 1.58  (1.31-1.91) | **< 0.001** | |

|  |  | **All Patients** | | | | | | | | | |
| --- | --- | --- | --- | --- | --- | --- | --- | --- | --- | --- | --- |
|  |  | TIC Component Normal-Abnormal Threshold | | | | | TIC Score Prediction of Outcome Measure | | | | |
|  |  | Clot  Time | Alpha  Angle | Maximum Amplitude | Total Possible  TIC Score | Any Abnormal Value (TIC+)  n (%) | Mortality | | | pRBC Transfusion Volume | |
|  |  |  |  |  |  |  | OR  (95% CI) | p-value | AUROC | IRR  (95% CI) | p-value |
| **Model Permutation** | 1 | Baseline | Baseline | Baseline | 3 | 591  (39.4) | 1.53  (1.33-1.76) | **< 0.001** | 0.801 | 1.25  (1.16-1.34) | **< 0.001** |
|  | 10 | 2 SD  Less Extreme | Baseline | Baseline | 3 | 953  (63.5) | 1.58  (1.39-1.80) | **< 0.001** | 0.804 | 1.27  (1.19-1.36) | **< 0.001** |
|  | 11 | 1 SD  Less Extreme | Baseline | Baseline | 3 | 818  (54.6) | 1.54  (1.34-1.77) | **< 0.001** | 0.801 | 1.26  (1.17-1.35) | **< 0.001** |
|  | 12 | 1 SD  More Extreme | Baseline | Baseline | 3 | 559  (37.3) | 1.55  (1.34-1.80) | **< 0.001** | 0.799 | 1.28  (1.19-1.38) | **< 0.001** |
|  | 13 | 2 SD  More Extreme | Baseline | Baseline | 3 | 557  (37.2) | 1.56  (1.34-1.81) | **< 0.001** | 0.800 | 1.29  (1.19-1.39) | **< 0.001** |
|  | 14 | Baseline | 2 SD  Less Extreme | Baseline | 3 | 1499  (100.0) | 1.85  (1.48-2.30) | **< 0.001** | 0.799 | 1.42  (1.27-1.59) | **< 0.001** |
|  | 15 | Baseline | 1 SD  Less Extreme | Baseline | 3 | 1390  (92.7) | 1.69  (1.40-2.07) | **< 0.001** | 0.798 | 1.37  (1.24-1.51) | **< 0.001** |
|  | 16 | Baseline | 1 SD  More Extreme | Baseline | 3 | 511  (34.1) | 1.65  (1.40-1.93) | **< 0.001** | 0.801 | 1.29  (1.18-1.40) | **< 0.001** |
|  | 17 | Baseline | 2 SD  More Extreme | Baseline | 3 | 508  (33.9) | 1.74  (1.45-2.09) | **< 0.001** | 0.801 | 1.35  (1.22-1.48) | **< 0.001** |
|  | 18 | Baseline | Baseline | 2 SD  Less Extreme | 3 | 1498  (99.9) | 1.87  (1.50-2.32) | **< 0.001** | 0.800 | 1.30  (1.16-1.46) | **< 0.001** |
|  | 19 | Baseline | Baseline | 1 SD  Less Extreme | 3 | 1264  (84.3) | 1.67  (1.40-2.00) | **< 0.001** | 0.800 | 1.30  (1.19-1.43) | **< 0.001** |
|  | 20 | Baseline | Baseline | 1 SD  More Extreme | 3 | 481  (32.1) | 1.64  (1.39-1.92) | **< 0.001** | 0.801 | 1.26  (1.15-1.37) | **< 0.001** |
|  | 21 | Baseline | Baseline | 2 SD  More Extreme | 3 | 463  (30.9) | 1.86  (1.54-2.25) | **< 0.001** | 0.803 | 1.30  (1.18-1.43) | **< 0.001** |

**Supplemental Digital Content Table 5:** TIC score predictive abilities for mortality and pRBC transfusion with alternative cut points. Each model permutation adjusts one TEG component’s normal-abnormal threshold by one or two standard deviations.

**Supplemental Digital Content Table 6:** Predictive abilities of TIC score and abnormal TEG components on mortality and pRBC transfusion volumes, *after excluding patients with missing ISS and utilizing ISS as a covariate*. Effect on mortality is presented as odds ratio, while pRBC transfusion is presented as an incidence rate ratio.

|  |  | **Outcome Measure** | | | |
| --- | --- | --- | --- | --- | --- |
|  |  | Mortality | | pRBC  Transfusion Volume | |
|  |  | OR  (95% CI) | p-value | IRR  (95% CI) | p-value |
| **Predictive Factor** | TIC Score | 1.46  (1.26-1.69) | **< 0.001** | 1.21  (1.13-1.29) | **< 0.001** |
|  | Clot Time Abnormal | 1.59  (0.99-2.55) | 0.053 | 1.02  (0.82-1.24) | 0.886 |
|  | Alpha Angle Abnormal | 1.46  (0.95-2.23) | 0.082 | 1.06  (0.88-1.27) | 0.566 |
|  | Maximum Amplitude Abnormal | 1.41  (0.92-2.16) | 0.118 | 1.49  (1.24-1.79) | **< 0.001** |
